# European Society for Organ Transplantation Consensus Statement on Biomarkers in Liver Transplantation

**DOI:** 10.3389/ti.2023.11358

**Published:** 2023-08-30

**Authors:** Marina Berenguer, Eleonora de Martin, Amelia J. Hessheimer, Josh Levitsky, Daniel G. Maluf, Valeria R. Mas, Nazia Selzner, Helena Hernàndez-Èvole, Alina Lutu, Nabeel Wahid, Haseeb Zubair

**Affiliations:** ^1^ Hepatology and Liver Transplantation Unit, Hospital Universitario la Fe - IIS La Fe Valencia, CiberEHD and University of Valencia, Valencia, Spain; ^2^ AP-HP Hôpital Paul Brousse, Centre Hépato-Biliaire, Inserm UMR-S 1193, Université Paris-Saclay, Villejuif, France; ^3^ General & Digestive Surgery, Hospital Universitario La Paz, Instituto de Investigación Hospital Universitario La Paz (IdiPAZ), Centro de Investigación Biomédica en Red de Enfermedades Hepáticas y Digestivas (CIBERehd), Madrid, Spain; ^4^ Division of Gastroenterology and Hepatology, Department of Medicine, Comprehensive Transplant Center, Feinberg School of Medicine, Northwestern University, Chicago, IL, United States; ^5^ Program in Transplantation, Department of Surgery, University of Maryland Medical Center, University of Maryland School of Medicine, Baltimore, MD, United States; ^6^ Surgical Sciences Research in Transplantation, Chief Surgical Sciences Division, Department of Surgery, University of Maryland School of Medicine, Baltimore, MD, United States; ^7^ Ajmera Transplant Center, Toronto General Hospital, University of Toronto, Toronto, ON, Canada; ^8^ Liver Unit, Hospital Clinic de Barcelona, Barcelona, Spain; ^9^ Surgical Sciences Division, University of Maryland School of Medicine, Baltimore, MD, United States

**Keywords:** liver transplantation, biomarkers, chronic kidney disease, hepatocellular carcinoma, rejection, recurrent primary diseases

## Abstract

Currently, one-year survival following liver transplantation (LT) exceeds 90% in large international registries, and LT is considered definitive treatment for patients with end-stage liver disease and liver cancer. Recurrence of disease, including hepatocellular carcinoma (HCC), significantly hampers post-LT outcomes. An optimal approach to immunosuppression (IS), including safe weaning, may benefit patients by mitigating the effect on recurrent diseases, as well as reducing adverse events associated with over-/under-IS, including chronic kidney disease (CKD). Prediction of these outcome measures—disease recurrence, CKD, and immune status—has long been based on relatively inaccurate clinical models. To address the utility of new biomarkers in predicting these outcomes in the post-LT setting, the European Society of Organ Transplantation (ESOT) and International Liver Transplant Society (ILTS) convened a working group of experts to review literature pertaining to primary disease recurrence, development of CKD, and safe weaning of IS. Summaries of evidence were presented to the group of panelists and juries to develop guidelines, which were discussed and voted in-person at the Consensus Conference in Prague November 2022. The consensus findings and recommendations of the Liver Working Group on new biomarkers in LT, clinical applicability, and future needs are presented in this article.

## Introduction

The consensus development process was organized by a dedicated Guidelines Taskforce within ESOT and its sections, which include ELITA, EKITA, EPITA, ECTTA, ETHAP, the Education Committee, YPT, Transplant International editorial board members and patient representatives. A detailed description of methodology used has been reported previously [1].

Briefly, key issues related to biomarkers in liver transplantation (LT) were identified by the Liver Working Group. Biomarkers were defined as characteristics that may be objectively measured and evaluated to serve as indicators of normal biological processes, pathological processes, or pharmacological responses to a therapeutic intervention. Specific clinical questions were formulated according to the PICO methodology (PICO = Population, Intervention, Comparator and Outcome). The four PICO questions related to disease recurrence, hepatocellular carcinoma (HCC) recurrence, chronic kidney disease (CKD), and weaning of immunosuppression (IS) are listed in Tables 1–4. Following the definition of PICOs, literature searches were conducted by expert staff from the CET (Centre for Evidence in Transplantation), who have expertise in conducting systematic reviews, and subsequently integrated, as needed, by the steering committee experts.

**TABLE 1:** Literature Search Request for the 4 PICO questions in Liver Transplantation. Recurrent disease in liver transplantation.

Topics and research questions	Can biomarkers be used to diagnose recurrent liver diseases (MASH, alcohol resumption, autoimmune diseases) after liver transplantation?
Population(s) e.g.:	
• Type of transplant(s)	Liver Transplantation
• Age (pediatric/adult)	Adult
• Condition	Patients with and without elevated liver enzymes
Intervention	Use of biomarkers to diagnose recurrent diseases
Comparators (Where appropriate)	Diagnosis of recurrent disease based on liver biopsy +/- imaging data
Outcomes	- Recurrent disease in the graft (MASH, ASH, AIH, PBC)
Exclusion criteria (optional)	1. Journal with IF <2
	2. Papers without a clear ethical approval
	3. Systemic review and metanalysis
	4. Conference abstracts
Search period	2000-day of the research
Types of studies	Randomized controlled trials
	Registry analyses
	Observational studies
Language	English
Comments/context/suggested keywords	- Liver transplantation
	- Recurrent liver disease
	- MASH
	- Alcohol relapse
	- Autoimmune diseases (autoimmune hepatitis, primary biliary cholangitis/primary sclerosing cholangitis recurrence)

**TABLE 2:** Literature Search Request for the 4 PICO questions in Liver Transplantation. Recurrent HCC in liver transplantation.

Topics and research questions	Can biomarkers be used to predict HCC recurrence
Population(s) e.g.:	
• Type of transplant(s)	Liver transplant recipients undergoing LT due to HCC disease
• Age (pediatric/adult)	Adult
• Condition	HCC
Intervention	Use of biomarkers to predict HCC recurrence and thereby improve posttransplant monitoring
Comparators (Where appropriate)	Prediction of HCC recurrence based on classical models (up to seven Model, Milan criteria, Retreat Model)
Outcomes	- HCC recurrence
	- Cost of post-transplant monitoring
	- HCC recurrence free survival
	- Post-transplant survival
Exclusion criteria (optional)	1. Journal with IF <2
	2. Papers without a clear ethical approval
	3. Systemic review and metanalysis
	4. Conference abstracts
Search period	2010–2022
Types of studies	Randomized controlled trials
	Diagnostic studies
	Observational studies
Language	English
Comments/context/suggested keywords. Please give as much detail as possible	- Evaluation of biomarkers (conventional and new ones)
	- Molecular biomarkers: gene expression, microRNAs, proteomics, metabolomics, cell free DNA, cell free methylated DNA, cell free RNA.
	- Non-invasive biomarkers in different sample types: peripheral blood mononuclear cells, plasma, serum
	- Biomarkers using liver graft tissue
	- Specificity, sensitivity, positive predictive value, negative predictive value
	- Gold standard, controls, study endpoints

**TABLE 3:** Literature Search Request for the 4 PICO questions in Liver Transplantation. Immunosuppression weaning in Liver Transplantation.

Topics and research questions	Can biomarkers be used to safely wean IS (minimization and eventually full withdrawal)?
Population(s) e.g.:	
• Type of transplant(s)	Liver transplant recipients receiving maintenance immunosuppression.
• Age (adult)	Adult
• Condition	Maintenance IS
Intervention	Use of biomarkers to guide IS minimization and withdrawal
Comparators (Where appropriate)	IS minimization and withdrawal based on classical clinical approach (risk factors associated with rejection, time from LT, trough levels)
Outcomes	- weaning IS without rejection
	- time to minimal/no immunosuppression
	- adverse events associated with IS (Diabetes, AHT, CVD, *de novo* cancer), subclinical graft injury
	- acute rejection
Exclusion criteria (optional)	1. Journal with IF <2
	2. Systemic review and metanalysis
	3. Conference abstracts
	4. Studies with less than 25 patients
Search period	January 2005- May 2022
Types of studies	Randomized controlled trials
	Diagnostic studies
	Observational studies
Language	English
Comments/context/suggested keywords	- Evaluation of biomarkers (conventional and new ones)
	- Molecular biomarkers: gene expression, microRNAs, proteomics, metabolomics, cell free DNA, cell free methylated DNA, cell free RNA.
	- Non-invasive biomarkers in different sample types: peripheral blood mononuclear cells, plasma, serum
	- Biomarkers using liver graft tissue
	- Specificity, sensitivity, positive predictive value, negative predictive value
	- Gold standard, controls, study endpoints

**TABLE 4:** Literature Search Request for the 4 PICO questions in Liver Transplantation. Chronic kidney disease development in liver transplantation.

Topics and research questions	Can biomarkers be used to predict chronic kidney disease (CKD) in liver transplant recipients
Population(s) e.g.:	
• Type of transplant(s)	Liver transplant recipients receiving maintenance immunosuppression
• Age (pediatric/adult)	Adult
• Condition	Maintenance immunosuppression
Intervention	Use of biomarkers to predict future development of CKD and progression to end stage renal disease (ESRD)
Comparators (Where appropriate)	CKD prediction based on classical clinical approach (risk factors associated with CKD such as diabetes, hypertension, age, pre-LT kidney function, trough levels of calcineurin inhibitors…)
Outcomes	- Development of CKD stage III (<60 mL/min eGFR)
	- Progression through different stages of CKD (I to V)
	- Development of ESRD (CKD stage V), need for hemodialysis, need for kidney transplantation
	- Patient/graft survival in relation to CKD stage
Exclusion criteria (optional)	1. Journal with IF <2
	2. Papers without a clear ethical approval
	3. Systemic review and metanalysis
	4. Conference abstracts
Search period	January 2005- May 2022
Types of studies	Randomized controlled trials
	Diagnostic studies
	Observational studies
Language	English
Comments/context/suggested keywords. Please give as much detail as possible	- Evaluation of biomarkers (conventional and new ones)
	- Non-invasive biomarkers in different sample types: mainly plasma, serum, urine, DNA (genetic predictors)
	- Predictive models (clinical alone, biomarker alone, clinical + biomarker)
	- Specificity, sensitivity, positive predictive value, negative predictive value
	- Would include endpoints of GFR: serum creatinine-based estimated (eGFR) using MDRD, CKD-EPI; addition of cystatin-C to these equations; measured GFR using inulin, iothalamate, iohexol, or even radionuclide renal scans

The Working Group proposed a recommendation for each key question, based on the quality of evidence rated using the GRADE approach, with high quality rated as A, medium quality as B, and low quality as C, and very low quality of evidence as D. For evaluation of the quality of evidence according to GRADE, the following features were considered: study design, risk of bias, inconsistency, indirectness, imprecision, number of patients, effect, importance, and publication bias. Strength of recommendation was rated as 1 (strong) or 2 (weak).

## Recurrence of Liver Diseases After Liver Transplantation

Autoimmune diseases, such as primary sclerosing cholangitis (PSC), primary biliary cholangitis (PBC), and autoimmune hepatitis (AIH), represent about 8% of indications for LT [2], with 5-year patient survival rates surpassing 85% [3]. Disease recurrence, which is primarily responsible for impaired graft survival, is seen in 8.6%–27% of patients transplanted for PSC [4, 5], 10.9%–42.3% for PBC [6], and 7%–42% for AIH [7]. The diagnosis of recurrent disease is based on a combination of clinical, biological, and histological criteria and is often challenging [8].

Several studies are based on histology and even encourage performing protocol liver biopsy, which can facilitate the diagnosis of disease recurrence in the absence of biochemical and immunological abnormalities [9]. For recurrent PBC (rPBC), the gold standard for diagnosis is histological findings, including bile duct destruction by epithelioid granulomas, lymphocyte cholangitis, ductular proliferation, lymphocytic aggregates, and bile duct paucity. Elevated alkaline phosphatase and anti-mitochondrial antibody (AMA) levels are unreliable diagnostic markers [10]. For recurrent PSC (rPCS), diagnosis is made when cholangiographic imaging and liver biopsy findings similar to those described in native livers with PSC are observed in the context of mild cholestasis [11]. Pre-LT immunoglobulin G (IgG) level, high transaminase levels, severe inflammatory activity or plasma cell infiltration in the liver explant, concomitant autoimmune disease, recipient age <42 years, and donor-recipient sex mismatch have been associated with higher risk of recurrent AIH (rAIH) [12, 13]. Post-transplant auto-antibodies, such as anti-nuclear (ANA), anti-smooth muscle antibodies (ASMA), and anti-LKM at high titer, are also predictive of rAIH [14], even though they also appear in 64% of patients transplanted for non-autoimmune liver diseases and are therefore not specific [15]. Similar to the pre-LT setting, rAIH is characterized by elevated transaminases, hyper-gammaglobulinemia, and increased IgG. The gold standard for diagnosing disease recurrence remains histology, with typical features including lymphoplasmocytic interface hepatitis, lobular hepatitis, and portal plasmocytic infiltration [16, 17].

Metabolic dysfunction associated steatohepatitis (MASH) is one of the most frequent liver diseases in the United States and Europe [18], and its prevalence varies from 7% to 30% among metabolic dysfunction-associated steatotic liver disease (MASLD) patients [19]. MASH has become the second indication for LT after alcohol-related liver disease in the United States (US), and it currently represents 8.4% of LT indications in Europe [20]. In terms of post-transplant outcomes, 10-year graft survival of 62% has been described, similar to non-MASH patients [21]. On the contrary, another study from the United States described post-LT graft survival that was significantly lower compared to PSC, PBC, and AIH indications [22]. Pre-transplant factors, such as metabolic syndrome, insulin resistance, and arterial hypertension, are not reliable predictors of disease recurrence. Rather, high pre- and post-LT body mass index and increased post-LT triglyceride levels were significant predictors [23]. Simliar to autoimmune liver diseases, liver biopsy remains the most reliable method for assessing rMASH and its severity after LT [24, 25].

For all of the aforementioned diseases, liver biopsy remains the gold standard for the diagnosis of primary disease recurrence. As such, identification of more reliable biomarkers is urgently needed.

### Methods

**FIGURE 1:**
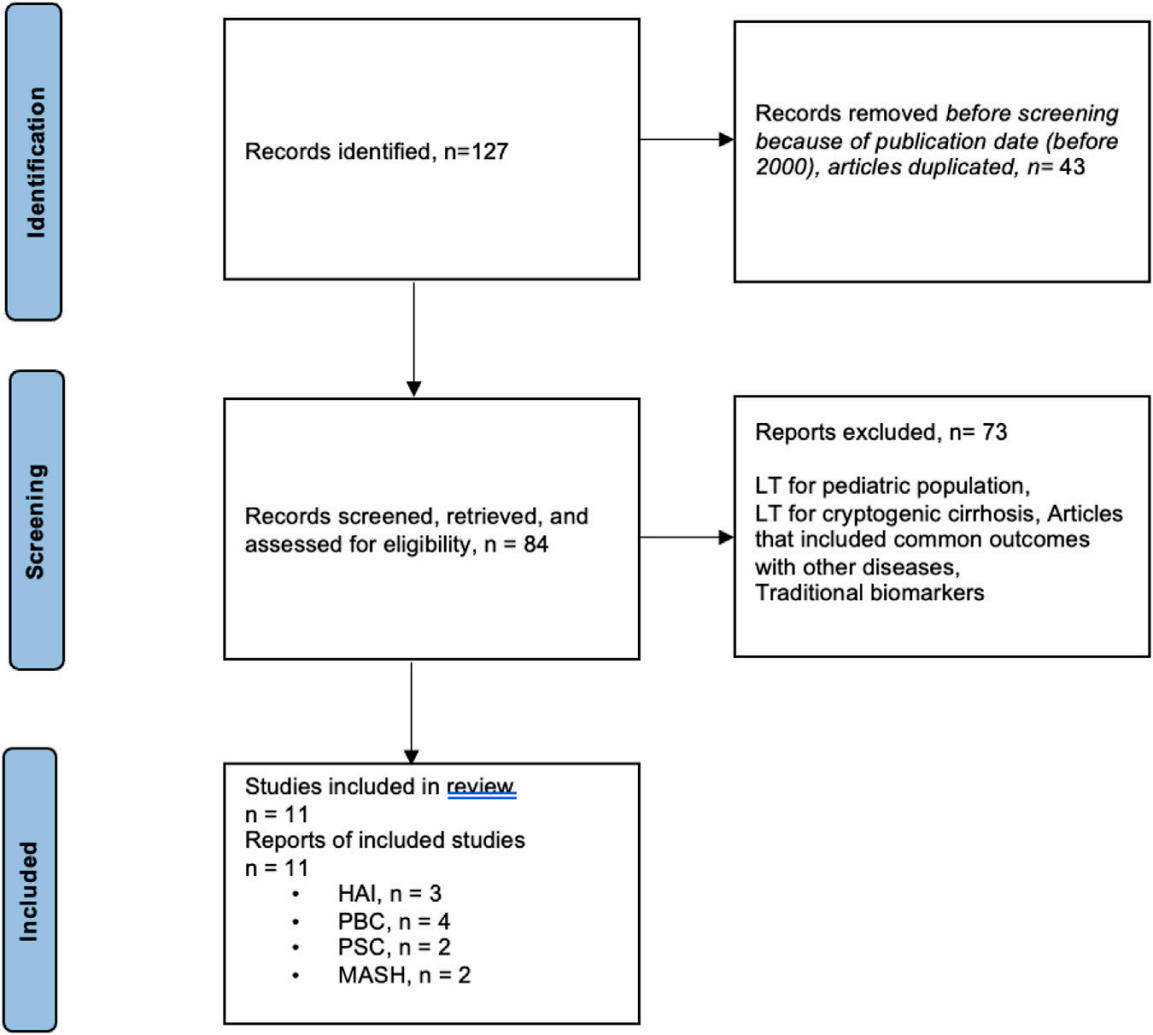
Flowchart summarizing the selection process of studies included in the evaluation of biomarkers for recurrent diseases in liver transplantation.

MEDLINE and EMBASE databases were used to search for relevant articles (Table 1). The following search terms were used in the MEDLINE database: liver transplantation/recurrent liver disease/MASH/autoimmune diseases (autoimmune hepatitis, primary biliary cholangitis)/primary sclerosing cholangitis recurrence. A manual search was also conducted of the reference lists in the review articles. The study inclusion period was 2000–2022. Prospective, observational, and diagnostic studies and reviews were included. Specific exclusion criteria were (i) studies including LT for cryptogenic disease, even though autoimmune liver diseases or MASH were diagnosed on follow up; (ii) studies including clinical parameters such as hypertension; body mass index; or classic biological parameters, such as liver enzymes, bilirubin, alkaline phosphatase, AMA, ANA, ASMA, IgG, serum glucose, HbA1c, cholesterol, and/or triglycerides. The flowchart summarizing the literature search is reflected in Figure 1.

### Results

A total of 127 articles were found on recurrent primary diseases, and 11 studies were selected (Supplementary Table S1): 3 studies for AIH, 4 for PBC, 2 for PSC, and 2 for MASH. The aims of the studies were to evaluate risk factors for disease recurrence. Eight out of 9 studies reflected the role of human leukocyte antigen (HLA) as risk factor for recurrent autoimmune diseases. The study of Gonzalez-Koch et al. demonstrated that HLA-DR3 or HLA-DR4 with HLA-DR3 were more important risk factors for rAIH than HLA-DR4, even though the difference was not statistically significant [26]. Another study identified HLA-DR3 phenotype in the recipient and/or donor as a risk factor for rAIH [17]. More recently, high-level HLA-DR mismatch was associated with an increased risk of rAIH [27]. Concerning rPBC, in Sanchez’s study, only donor alleles A1, B57, B58, DR44, DR57, and DR58 and recipient allele B48 were found more frequently in patients with disease recurrence, but there was no significant association for HLA mismatches between donor and recipient [28]. On the other hand, Guy et al. found an increased mismatch of donor DR3 and recipient DR4 in patients with rPBC [29], and another study reported that HLA-A, -B, and -DR mismatches were risk factors for disease recurrence [30]. The study from Carbone et al. found that risk of rPBC was greatest for rs62270414 genotype for IL12A locus [31].

Regarding rPSC, in one study which had all HLA data available for all donors and recipients, HLA-DRB1*08 allele was detected in either donor or recipient with rPSC [32]. On the other hand, in the study by Bajer et al., HLA-DRB1*07 in the donor represented a potential risk factor for rPSC [33]. For rNASH, G-allele in position rs738409 of patatin-like phospholipase domain-containing protein 3 (PNPLA3) presence in the recipient was associated with an increased hepatic concentration of triglycerides and with rMASH, though liver biopsy to confirm the diagnosis was only available in a minority of patients and recurrent disease diagnosis was based on biological and clinical criteria [34]. A more recent study found 16 metabolites associated with rMASH compared to MASLD. The most differentially expressed chemical class was phosphatidylcholines, with 10 of these lipids significantly decreased in the MASH cohort. The remaining metabolites consisted of AAs, sterols, phosphatidylethanolamines, and phingomyelins [35].

The summary of the evidence addressing the recurrent diseases in LT key question by included studies is shown in Table 5.

**TABLE 5:** Summary of evidence for biomarkers in recurrent diseases after LT.

Number of studies			No. of patients	Factors that may decrease the certainty of the evidence					Quality of evidence (GRADE)
RCT	Observational comparative	Observational non-comparative		Risk of bias	Indirectness	Inconsistency	Imprecision	Publication bias	
Index Test 1: AIH recurrence									
0	0	3	133	serious	not serious	not serious	serious	Likely	Very Low (D)
Index Test 2: PBC recurrence									
0	4	0	502	serious	serious	very serious	serious	Likely	Very Low (D)
Index Test 3: PSC recurrence									
0	1	1	116	serious	serious	serious	serious	Likely	Very Low (D)
Index Test 4: MASH recurrence									
0	0	2	274	serious	serious	very serious	serious	Likely	Very Low (D)

Effect estimates from comparative studies: This is a qualitive (not quantitative) evaluation of the effect estimate/size derived from comparative studies. Examples are shown above on such assessments. Limitations: Make a judgement on the risk of bias across studies for an individual outcome. It is possible to consider the size of a study, its risk of bias and the impact it would have on the summarized effect. Inconsistency: Evaluate the difference in the magnitude of effects across studies. Widely differing estimates of the effects indicate inconsistency. Indirectness: Make a global judgement on how dissimilar the research evidence is to the clinical question at hand (in terms of population, interventions, and outcomes s studies). Imprecision: Consider the optimal information size (or the total number of events for binary outcomes and the number of participants in continuous outcomes) across all studies. Results may also be imprecise when the confidence intervals (CI) of all the studies or of the largest studies include no effect and clinically meaningful benefits or harms. Publication bias can be suspected when the body of evidence consists of only small positive studies or when studies are reported in trial registries but not published. Statistical evaluation of publication bias is not possible in this case.

### Recommendation

Additional studies are needed before any recommendation can be issued regarding the application of biomarkers to reliably predict and/or diagnose disease recurrence after liver transplantation.

Quality of evidence: Very Low.

Grade of recommendation: Strong for.

### Discussion and Next Steps

Post-LT recurrence of the initial disease process is heterogeneous in presentation and severity. Due to its impact on long-term outcomes, it is important to identify new biomarkers for early identification.

Among the 9 studies selected for autoimmune diseases, the majority had a small sample size, with only two studies including more than 100 patients. The small cohorts can be explained by the rarity of these recurrent diseases. Eight studies supported specific HLA or donor-recipient HLA mismatches as risk factors for disease recurrence. However, given the small number of patients included and the differences in disease diagnosis (per protocol versus clinically indicated liver biopsy), the correlation between HLA and recurrent autoimmune diseases should be further investigated, and strong recommendations cannot be made. One study evaluated genetic loci associated with rPBC. Though the study was well-conducted on a relatively large cohort of patients, it remains singular, and more data are needed.

Regarding rMASH, metabolomic analysis was shown in one study to be a promising tool. Further studies are needed, however, as the study included a small number of observations and analyzed many variables, thereby increasing the potential for errors.

Overall, given the low number of studies addressing this issue and their retrospective nature, the small number of patients included, heterogeneous inclusion criteria and results, and incomplete datasets in some instances, no strong recommendations regarding the use of specific biomarkers to detect post-LT recurrence of primary liver disease can be made. Prospective studies must be conducted to establish the role of biomarkers in predicting and diagnosing these processes.

## Recurrence of Hepatocellular Carcinoma After Liver Transplantation

Hepatocellular carcinoma (HCC) is one of the most common cancers worldwide, with an incidence that is predicted to increase in the coming decades [36]. Unfortunately, mortality associated with HCC remains high. In fact, treatment strategies are only curative for early-stage tumors. Among these, LT is considered the best treatment option for BCLC A stage one patients selected according to Milan criteria (MC) [37]. Although application of MC led to a significant decrease in recurrence rates, recurrence still occurs in some that fulfill the criteria and, more importantly, leaves out a significant proportion who might be cured by LT despite being outside MC [38]. Several models have been proposed to expand LT HCC inclusion criteria, usually based on morphological features, simple biological markers (e.g., alpha-fetoprotein—AFP), explant pathology, and/or response to locoregional therapy (LRT). Depending on the time frame they are applied (pre-vs. post-LT), they might be used to predict recurrence and help in the selection process and/or to adapt post-LT strategies. These models have shown to adequately predict recurrence risk, yet they continue to lack molecular factors reflecting the biological complexity of HCC and remain only partially predictive in this regard [38, 39].

Indeed, there are many known genetic mutations and other molecular alterations occurring in HCC tumors, and multiple studies report associations between molecular biomarkers and tumor-specific post-LT outcomes (i.e., presence, timing, and location and/or extent of HCC recurrence) [40]. Biomarkers that have been assessed in human tissue appear useful for the classification of HCC into subclasses indicative of disease aggressiveness and prognosis. While theoretically promising, drawbacks associated with such assays and biologically based classification systems include lack of prospective, well-powered studies that definitively establish their ability to accurately predict post-LT HCC recurrence and/or survival [41, 42]. As well, molecular assays relying on tissue are invasive and often require the actual liver explant for their assessment, severely (if not altogether) limiting their utility in pre-LT patient stratification and selection and optimization of liver allograft utilization (primary goals). “Liquid biopsy” is promising tool in this regard, as it represents a minimally invasive approach to analyzing tumor components (cells or small pieces of DNA, RNA, or other molecules released by tumor cells) without need for tissue [43–45]. Liquid biopsy is dynamic and may be assessed at different peri- and post-LT time points [46].

### Methods

The specific question that was made for literature review was (Table 2): Can circulating tumor biomarkers be used to predict HCC recurrence? The study population included adult liver transplant recipients undergoing LT due to HCC related liver disease. The intervention was the analysis of whether circulating tumor cells or components could accurately predict HCC recurrence and thereby improve posttransplant monitoring, while the comparator was the use of classical models (up-to-seven, Milan criteria, RETREAT Model). Outcomes assessed included HCC recurrence, cost of post-transplant monitoring, HCC recurrence-free survival, and overall post-transplant survival.

The initial literature search was performed by the CET, followed by the inclusion of additional articles extracted from the bibliographies. The study period was 2010–2022. Inclusion criteria were English language studies published on adult patients (18 years and older) analyzing the association between circulating tumor biomarkers and post-LT HCC recurrence. Exclusion criteria included evaluation of traditional serum biomarkers (AFP, serum C-reactive protein, des-gamma-carboxy prothrombin, bilirubin, lipid profile, and protein induced by vitamin K absence or antagonist-II) as well as tissue-based biomarkers. Randomized clinical trials, diagnostic, and observational studies were included.

### Results

**FIGURE 2:**
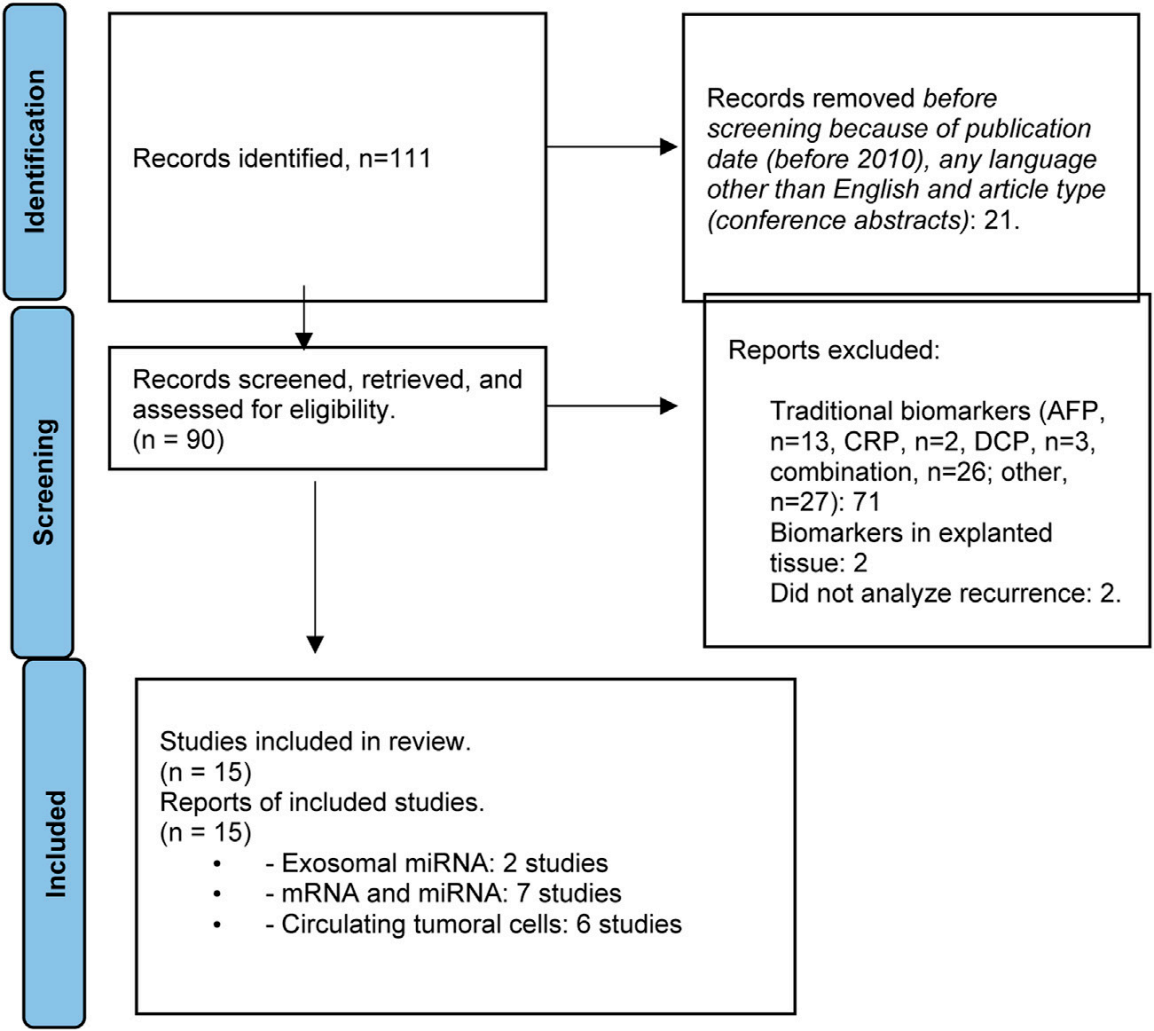
Flowchart summarizing the selection process of studies included in the evaluation of biomarkers for HCC recurrence.

The literature search produced a total of 111 articles. Excluding publications arising prior to 2010, those written in a language other than English, congress publications, articles addressing traditional biomarkers or biomarkers evaluated in explanted tissue, and studies in which detecting HCC recurrence was not the objective, a total of 15 studies related to liquid biopsy were included. The PRISMA flowchart describing the number of studies identified by the literature search and number of studies selected for inclusion in the consensus statement appears in Figure 2.

According to the results of the literature search, few studies evaluating the utility of liquid biopsy for the assessment of HCC tumor biology in the LT setting, including risk for recurrence, have been published to date (Supplementary Table S2). Risk that negative results or studies have not been reported remains. Studies that have been published to date have focused on exosomal miRNA (2 studies) [47, 48], circulating messenger and micro-RNA (mRNA and miRNA, respectively) (7 studies) [49–55], and circulating tumor cells (CTCs) (6 studies) [53, 56–60].

Assessment of HCC CTCs in the LT setting has been reported in eight studies, though only six evaluate their prognostic value and relevance to post-LT outcomes. Studies evaluate CTCs at different peri-operative time points (both prior to and following LT) and include relatively small patient cohorts largely recruited in Asia. Data remain conflicting regarding the utility of isolated CTC measurements (pre-LT only, for example) in predicting HCC recurrence [56–58], while dynamic CTC assessment, including evaluation of CTC subtypes, may increase CTC prognostic capacity [53, 59, 60]. In general, while preliminary evidence appears to support a role for CTCs in HCC prognostication in LT candidates and recipients, larger prospective studies recruiting more patients in more geographical regions are needed before any recommendations regarding their use can be made.

Micro RNAs are short, non-coding RNAs that post-transcriptionally regulate gene expression by binding with mRNA; circulating levels of both have been measured in HCC LT recipients in the context of two and five studies, respectively. The preliminary results they report suggest potential associations between HCC recurrence and circulating pre-LT mRNAs encoding different proteins (albumin, h-TERT, AFP) as well as between HCC recurrence and circulating post-LT miRNAs [49–51, 54, 55]. Exosomal RNAs and circulating free DNA have also been evaluated in preliminary clinical studies and variably associated with post-LT recurrence and survival [47, 48].

The summary of the evidence addressing the HCC key question by the included studies is shown in Table 6.

**TABLE 6:** Summary of evidence for biomarkers in HCC after LT.

Number of studies			No. of patients	Factors that may decrease the certainty of the evidence					Quality of evidence (GRADE)
RCT	Observational comparative	Observational non-comparative		Risk of bias	Indirectness	Inconsistency	Imprecision	Publication bias	
Index Test 1: HCC recurrence									
0	4	10 (retrospective)	1,018	serious	serious	serious	serious	Likely	Very Low (D)
Index Test 2: Cost of posttransplant monitoring									
0	0	0							Very Low (D)
Index Test 3: HCC recurrence free survival									
0	2	6 (retrospective)	353	serious	serious	serious	serious	Likely	Very Low (D)
Index Test 4: Post-transplant patient survival									
0	0	3 (retrospective)	194	serious	serious	very serious	serious	Likely	Very Low (D)

Inconsistency: Evaluate the difference in the magnitude of effects across studies. Widely differing estimates of the effects indicate inconsistency. Indirectness: Make a global judgement on how dissimilar the research evidence is to the clinical question at hand (in terms of population, interventions, and outcomes across studies).

Imprecision: Consider the optimal information size (or the total number of events for binary outcomes and the number of participants in continuous outcomes) across all studies. Results may also be imprecise when the confidence intervals (CI) of all the studies or of the largest studies include no effect and clinically meaningful benefits or harms.

Publication bias can be suspected when the body of evidence consists of only small positive studies or when studies are reported in trial registries but not published. Statistical evaluation of publication bias is not possible in this case.

### Recommendation

In summary, on the question “Can biomarkers be used to predict HCC recurrence following liver transplantation,” and based on the low quality of evidence, the following recommendation was issued: While preliminary studies suggest a role for molecular biomarkers measured in liquid biopsy (circulating tumor cells, in particular) in prediction of HCC recurrence, additional studies are needed before any recommendation can be issued regarding their application in clinical practice, either as predictive factors to select patients for liver transplantation or to guide post-transplant management.

Quality of Evidence Low (C).

Strength of Recommendation Weak for.

### Discussion and Next Steps

HCC is one of the most common cancers worldwide and one of the most frequent indications for LT. Despite careful selection using MC, HCC recurs in some patients who meet criteria, and other patients are left out who could potentially benefit from this therapy. Currently, there are models mostly based on clinical variables and traditional biomarkers that predict recurrence and thus help with patient selection [38, 39]. Because many of the genetic alterations in HCC are now known and some have been associated with post-transplant outcomes, we aimed at determining the role of the new biomarkers in predicting HCC recurrence. The purpose of the present review was to evaluate the evidence for new biomarkers, and to determine their potential role in patient selection as well as recurrence surveillance. Our findings indicate that while there is potential to better select HCC patients, the evidence remains low, and these biomarkers cannot be recommended in clinical practice until more evidence is gathered.

Aside from the clear objective of improving candidate selection when applied in the pre-LT setting, a role for HCC molecular biomarkers in directing post-LT patient management is also discussed. Post-LT strategies that might be applied in high-risk patients include implementation of adjuvant systemic therapy/-ies and/or HCC surveillance protocols, though neither has been shown to be of clear clinical benefit [61]. As well, it is important to note that serial liquid biopsies performed during post-LT follow-up may create the complex and potentially distressing situation whereby HCC recurrence is “detected” (likely present) yet not located or visualized on cross-sectional imaging. How often such cases will arise and how best to proceed when they are encountered, with options including watchful waiting vs. “blind” administration of systemic therapy (both of which are associated with certain drawbacks for patients and clinicians) remain uncertain.

## Immunosuppression Weaning in Liver Transplantation

Outcomes following LT have significantly improved over the past three decades, and the use of modern immunosuppressant agents has been an important factor in this regard [62]. Unfortunately, the need for long-term IS is associated with serious complications and increases the chances of toxicities, rates of opportunistic infection, and malignancy [62–64]. For example, the use of CNIs increases the incidence of chronic kidney disease (CKD) in LT recipients [65]. Therefore, the establishment of long-term graft tolerance without ongoing need for IS is a primary goal in transplantation. However, we currently lack the tools necessary to identify patients who may benefit from IS minimization and withdrawal or to even identify those patients who are at risk of acute rejection (AR) upon IS reduction. Recent literature has described a variety of molecular, cellular, and histological markers originating from the peripheral blood and allograft that may help predict post-LT patients who can successfully be weaned off IS or who might be at risk of AR upon IS reduction. Although graft biopsy is an invasive technique and current practices are more interested in non-/minimally invasive techniques for patient stratification, we included graft biopsy-based biomarkers in our analysis, to assess if they offer any superior outcome compared to the recent “liquid biopsy” technique.

### Methods

**FIGURE 3:**
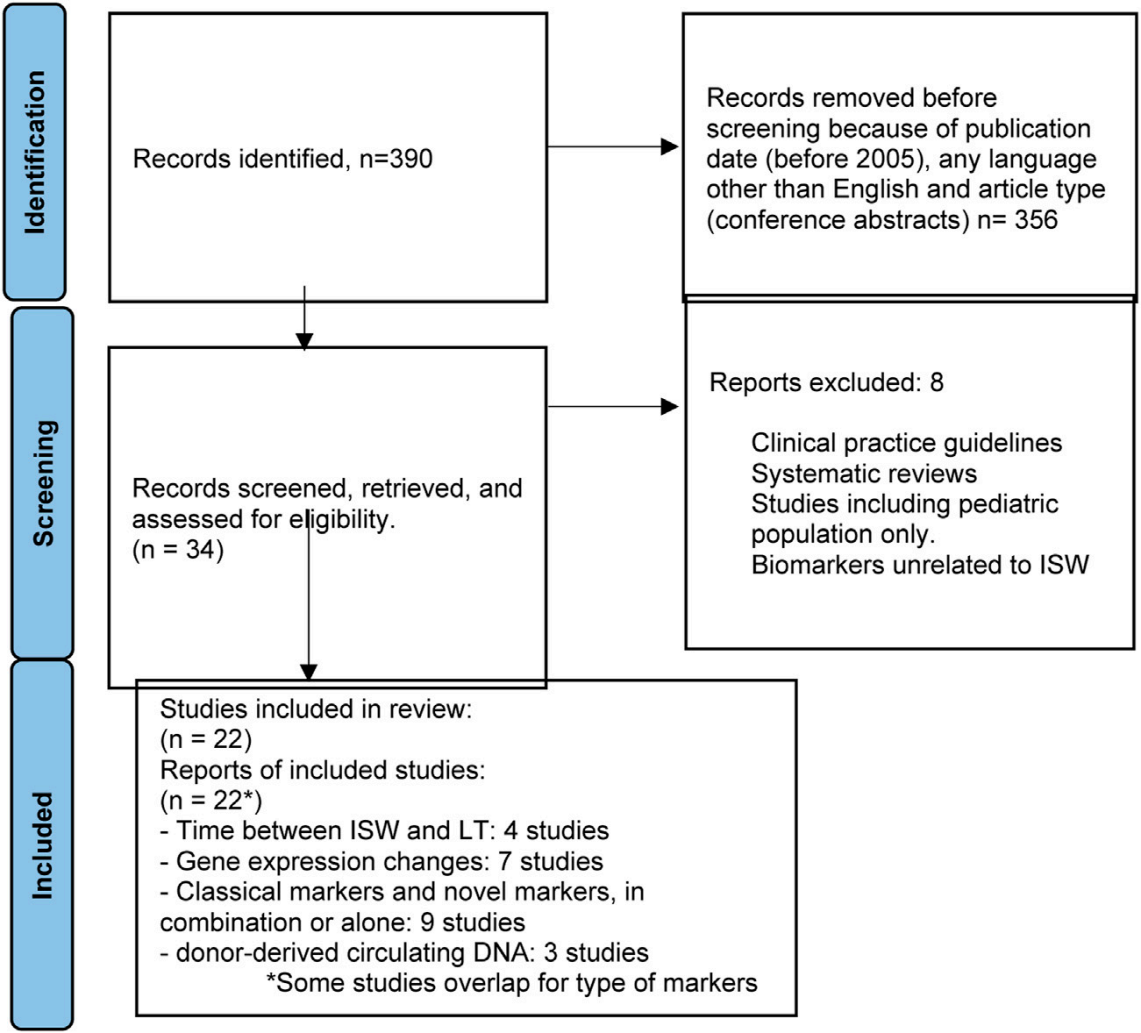
Flowchart summarizing the selection process of studies included in the evaluation of biomarkers for immunosuppression minimization and/or full withdrawal.

For the third PICO question, “Can biomarkers be used to safely wean IS (minimization and/or full withdrawal)?”, the study population was again adult liver transplant recipients undergoing IS minimization or withdrawal (Table 3). The population also consisted of patients who were assessed for markers for acute graft injury following LT. The outcome of the study was evaluation of non-invasive and invasive biomarkers from peripheral blood mononuclear cells, plasma, serum, and liver graft tissue. Molecular biomarkers of interest included gene expression, miRNAs, proteomics, metabolomics, cell-free DNA (cfDNA), cell-free methylated DNA, and cell-free RNA. The flowchart summarizing the literature search is reflected in Figure 3.

### Results

Supplementary Table S3 summarizes the studies assessing the role of biomarkers in safe IS minimization or withdrawal [66–87]. A positive association was observed in three studies between time from LT to IS withdrawal (ISW) among non-viral patients [66]. However, this remains conflicting, as North American studies have not observed this finding [87]. *De novo* donor specific antibody development was found to be associated with ISW [69]. Intra-tissue gene expression and immune cell infiltrations have been observed to have an association with induction and achievement of ISW. These, however, are invasive biomarkers and do not constitute effective biomarkers, and the studies supporting their use are potentially biased, as they were performed on relatively small numbers of patients.

Serum miRNA signatures were analyzed as biomarkers predicting development of operational tolerance (OT), with miR-483-3p and miR-885-5p signatures found to be positively associated with OT [70]. In transplantation, the contribution of donor-derived cfDNA has been an important indicator of graft injury post-transplantation [5, 84, 86]. Methylation-induced alterations in the released DNA were identified using droplet-digital PCR (ddPCR) to determine acute injury [82]. ddPCR was also used to identify genomic SNPs between donor and recipient to give a better indication of injury [86]. The donor-derived cfDNA (dd-cfDNA) had serial elevation of dd-cfDNA between injury and rejection and could identify pre-clinical graft injury in the context of normal liver function tests compared to rejection [83]. While these studies indicate the prospects of a non-invasive biomarker, independent validation and replication is needed using larger cohorts of LT patients from a variety of geographical and racial background to identify the benefit of their use.

The summary of the evidence addressing the IS minimization/withdrawal key question by the included studies is shown in Tables 7–9.

**TABLE 7:** GRADE approach-based summary of the quality of evidence for the development of operational tolerance or risk of injury upon weaning of immunosuppression.

Number of studies			No. of patients	Factors that may decrease the certainty of the evidence					Quality of evidence (GRADE)
RCT	Observational comparative	Observational non-comparative		Risk of bias	Indirectness	Inconsistency	Imprecision	Publication bias	
Index Test 1: Time between ISW and LT in non-viral patients									
0	3	0	163	not serious	not serious	serious^a^	not serious	None	Low (C)
Index Test 2: Combination of non-invasive PBMC GEX: FGL2/IFNG ratio and invasive baseline intrahepatic FOXP3/IFNG ratio at transplant									
0	0	1	14	serious^b^	not serious	not serious	not serious	None	Very Low (D)
Index Test 3: dnDSA during IS minimization									
0	2	0	130	not serious	not serious	serious^c^	not serious	None	Very Low (D)
Index Test 4: serum miRNA profile of hsa-miR-483-3p and hsa-miR-885-5p									
0	1	0	64	not serious	not serious	not serious	not serious	None	Low (C)
Index Test 5: Association between portal vein infiltrates and elapsed time post-ISW									
0	0	1	18	serious^b^	serious^d^	not serious	not serious	None	Very Low (D)
Index Test 6: Association of intrahepatic GEX of select genes^e^ and elapsed time post-ISW									
0	0	1	18	serious^b^	serious^d^	not serious	not serious	None	Very Low (D)
Index Test 7: *Ex vivo* cytokine production by PBMCs									
0	1	0	24	serious^b^	serious^f^	not serious	not serious	None	Very Low (D)
Index Test 8: Peripheral blood Vδ1/Vδ 2 T cell ratio quantification									
0	2	0	34	serious^b^	not serious	not serious	not serious	None	Low (C)
Index Test 9: Gender									
0	1	0	98	serious^b^	not serious	not serious	not serious	None	Low (C)
Index Test 10: Intrahepatic gene expression^g^									
0	1	0	75	serious^b^	serious^h^	not serious	not serious	None	Low (C)
Index Test 11: Serum hepcidin and ferritin									
0	1	0	80	serious^b^	not serious	not serious	not serious	None	Low (C)
Index Test 12: T-cell production of IFN-γ									
0	1	0	24	serious^b^	not serious	not serious	not serious	None	Low (C)

a While 4 studies report the benefit of a longer time duration between LT and ISW commencement, some studies did not find it significant in their patient cohort. Moreover, 2 of the studies use the same patient population.

^b^ Only one study with low sample size was included.

^c^ The cut-off for DSA MFI is not truly defined and different studies have used different MFI cut-offs, depending on the variability of mismatched HLA loci.

^d^ The invasive nature of the identified biomarker and post-ISW biopsy as indicators of graft acceptance are not efficient biomarkers for ISW-associated graft injury.

^e^ Genes of interest: FOXP3, CXCL10, CXCL9, UBD, IRF1, STAT1, IL32, CD52, CD68, STAT1, GPNMB, S1PR1, RGS5, ENPP2, MSL3, OPN3, PAK2, CDH5, SELP.

^f^ While the study demonstrates an increase in cytokine production, the isolation and culturing of PBMCs *ex vivo* will add complexity and is an indirect indicator of OT.

^g^ 5-gene (*CDHR2*, *MIF*, *PEBP1*, *SOCS1*, *TFRC*) signature and iron metabolism genes, *HAMP* and *TFRC* (FDR = 0, FC > |2|), and *FTHL12* and *FTHL8.*

^h^ Invasive biomarker and hence an indirect indicator of rejection.

**TABLE 8:** GRADE approach-based summary of the quality of evidence for the identification of subclinical graft injury and acute injury markers during IS.

Number of studies			No. of patients	Factors that may decrease the certainty of the evidence					Quality of evidence (GRADE)
RCT	Observational comparative	Observational non-comparative		Risk of bias	Indirectness	Inconsistency	Imprecision	Publication bias	
Index Test 1: Intrahepatic 11-gene marker for probable TCMR									
0	1	0	341	not serious	not serious	not serious	not serious	None	Low (C)
Index Test 2: Combination of ALT with liver stiffness measurement or DSAs									
0	0	1	185	serious^a^	serious^b^	not serious	not serious	None	Very Low (D)
Index Test 3: Combination of ALT with class II DSAs									
0	0	1	157	serious^a^	serious^b^	not serious	not serious	None	Very Low (D)
Index Test 4: serum miRNA profile of hsa-miR-483-3p and hsa-miR-885-5p									
0	1	0	130	not serious	not serious	not serious	not serious	None	Low (C)
Index Test 5: Galectin-1									
0	1	0	45	serious^a^	not serious	not serious^c^	not serious	None	Very Low (D)

^a^ Only one study with low sample size was included.

^b^ Study indicates the indirect stratification of patients with a medium to moderate injury.

^c^ Presentation of the data in the original article is a bit convoluted with the convention of naming of groups and sample size.

**TABLE 9:** GRADE approach-based summary of the quality of evidence for the genomic markers of acute injury post-liver transplantation.

Number of studies			No. of patients	Factors that may decrease the certainty of the evidence					Quality of evidence (GRADE)
RCT	Observational comparative	Observational non-comparative		Risk of bias	Indirectness	Inconsistency	Imprecision	Publication bias	
Index Test 1: detection of donor-derived cell-free DNA (dd-cf-DNA) using next-generation sequencing									
0	1	0	219	not serious	not serious	not serious	not serious	None	Moderate (B)
Index Test 2: detection of pre-identified donor DNA polymorphisms in dd-cf-DNA using droplet digital PCR									
0	2	0	185	not serious	serious^a^	not serious	not serious	None	Low (C)
Index Test 3: serum Diagnostic signature of miR-122 + miR210									
0	1	0	30	serious^b^	not serious	not serious	not serious	None	Low (C)
Index Test 4: plasma signature of miR-181a-5p									
0	1	0	145	not serious	not serious	not serious	not serious	None	Low (C)
Index Test 5: hepatocyte-specific methylated *PTK2B* as marker of dd-cf-DNA									
0	1	0	51	serious^b^	not serious	not serious	not serious	None	Low (C)

^a^ The two studies that have been identified using different cut-off values, thus reducing the potential assay adaptation.

^b^ Only one study with low sample size was included.

### Recommendation

Based on the moderate quality of evidence available, the following recommendation was issued: We suggest that biomarker assays may be able to help to guide ISW by monitoring liver injury. The use of longitudinal evaluations using non-invasive markers may lead to better stratification of patients for ISW.

Quality of evidence: Moderate.

Strength of recommendation: Weak for.

### Discussion and Next Steps

The prognostic and diagnostic value of invasive and non-invasive biomarkers to optimize IS and evaluate graft injury has been widely explored in LT. However, despite a decade of research, no LT biomarkers are currently available for use in clinical practice. Large multicenter clinical trials have generated vast amounts of data and information at various molecular levels, demonstrating a promising opportunity for cell-free biomarkers to be introduced into clinical care. Findings have not yet been translated into routine clinical use, due to small sample sizes, and the lack of proper control groups or independent validations.

## Chronic Kidney Disease (Ckd) in Liver Transplant Recipients

An estimated 40% of liver transplant recipients develop stage 3 CKD, and about 18% will develop end-stage renal disease within 5 years of LT, both of which are associated with increased risk of death [88, 89]. One of the primary culprits of renal deterioration post-transplant is calcineurin inhibitors. Although early reductions in CNIs within 1 year of transplant are associated with improvements in long-term renal function, reduced dosing of CNIs are also associated with higher rates of AR [90]. As such, identifying biomarkers for predicting CKD in liver transplant recipients would help select patients for early CNIs dose reductions and other nephroprotective interventions.

### Methods

**FIGURE 4:**
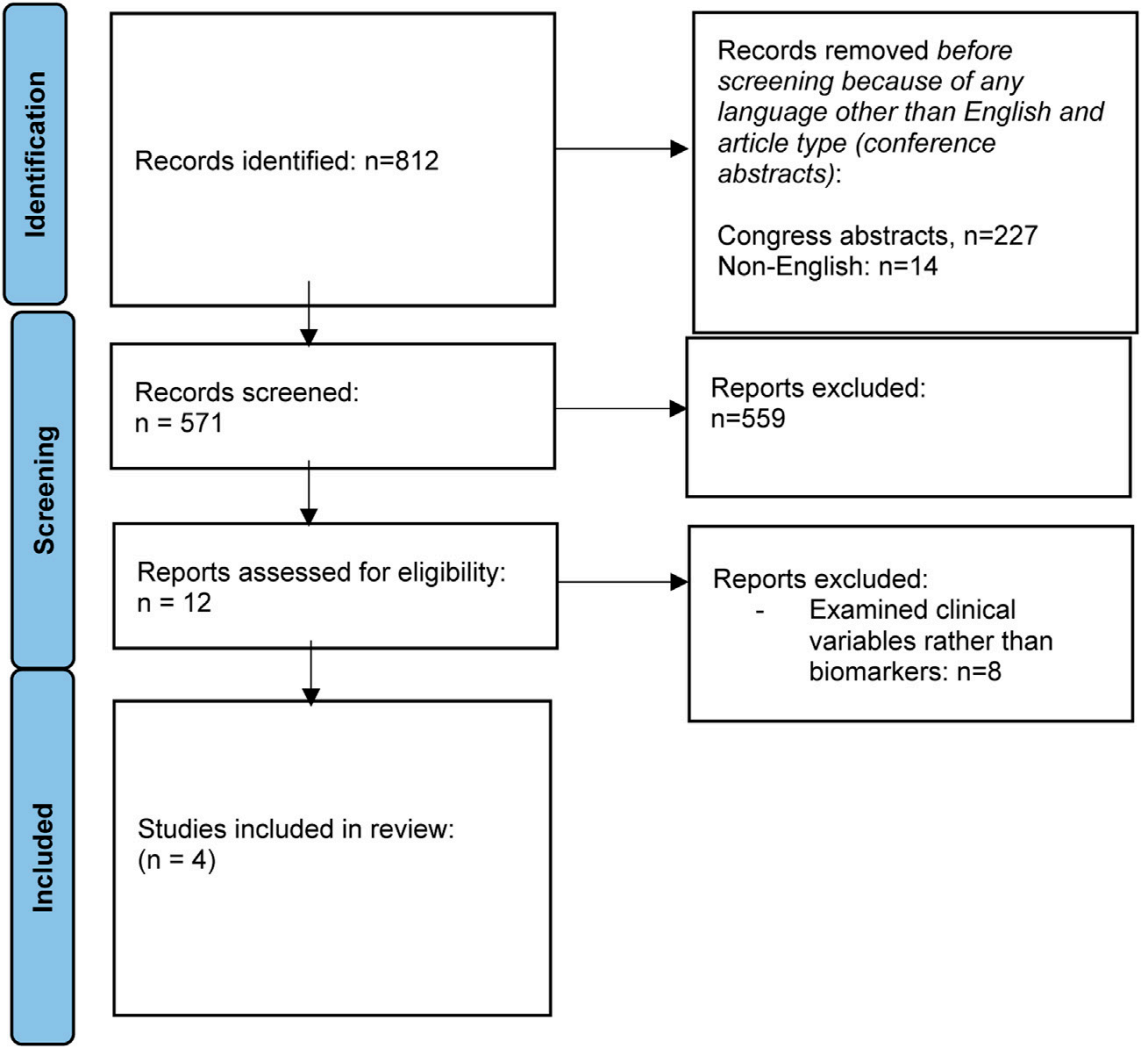
Flowchart summarizing the selection process of studies included in the evaluation of biomarkers for CKD after liver transplantation.

The search on the topic question “Can biomarkers be used to predict chronic kidney disease (CKD) in liver transplant recipients” is summarized in Table 4. Adult LT recipients under maintenance IS were the focus of the literature search. Outcome measures included (i) development of CKD stage III (<60 mL/min eGFR), (ii) progression through different stages of CKD (I to V); (iii) development of ESRD (CKD stage V), need for hemodialysis, need for kidney transplantation; and (iv) patient/graft survival in relation to CKD stage. The flowchart summarizing the literature search is reflected in Figure 4.

### Results

Most of the literature assessing variables associated with post-LT CKD examines clinical variables, rather than biomarkers. Only four studies were identified that assess the role of biomarkers in predicting post-transplant CKD [91–94]. Supplementary Table S4 summarizes baseline characteristics of the studies reviewed. PRESERVE used a discovery and validation cohort to develop a predictive model for post-LT CKD, incorporating beta-2 microglobulin (B2MG) and CD40 antigen [91]. Cullaro et al demonstrated that post-LT urinary neutrophil gelatinase-associated lipocalin (uNGAL) may be helpful in predicting post-LT CKD, particularly when combined with clinical variables [92]. Levitsky et al used proteomic testing to identify several proteins of interest, which may be associated with post-LT CKD [93], while Milongo et al found no association between the pre-LT urinary peptidome and CKD 6 months post-LT [94].

The summary of the evidence addressing the prediction of CKD among stable LT recipients is shown in Table 10.

**TABLE 10:** Summary of the evidence addressing the prediction of CKD.

Paper	Summary	Quality of evidence (GRADE)
Levitsky 2020	“PRESERVE”	Moderate (B)
	Analytic approach: Used discovery cohort to develop prediction model for GFR deterioration using 16 proteins in samples collected after LT; validated prediction model using validation cohort	
	Results: Developed predictive model using proteins including Beta-2 microglobulin (B2MG) and CD40 antigen; model had area under the curve (AUC) of 0.814 in discovery cohort and 0.801 in validation cohort year 1 GFR deterioration	
	Limitations: single sample collection timepoint; hepatitis C virus infection status included in predictive model (not a biomarker)	
Cullaro 2018	Analytic approach: receiver operating characteristic curves used to determine Urinary neutrophil gelatinase-associated lipocalin (uNGAL) cutoffs that maximized sensitivity/specificity	Low (C)
	Results: uNGAL at 24 h, 24-hour post-LT renal function, initial calcineurin inhibitor use, and age were independent predictors of CKD; AUC for uNGAL24h for CKD at 4 years was 0.65; when all the above variables combined in model- AUC 0.84 at 4 years post-LT	
	Limitations: single center; no validation cohort; incorporated non-biomarkers into predictive model (i.e., age, calcineurin inhibitor use, etc)	
Levitsky 2011	Analytic approach: retrospective identification of clinical characteristics associated with CKD in post-LT patients; proteomic testing in two independent cohorts (test and validation)	Low (C)
	Results: Age, cyclosporine use, and pre-LT GFR independently associated with new onset CKD; 10 proteins associated with new CKD in proteomic evaluations when GFR inputted as a continuous variable including: Cyc, alpha-1-microglobulin, beta-2-microglobulin, TFF3, FABP, factor VII, apolipoprotein H, apolipoprotein CIII, chromogranin A, and CD40 (notably NGAL was not associated with CKD)	
	Limitations: not a prospective study; single sample collection timepoint; single center	
Milongo 2015	Analytic approach: prospective study; pre-transplant urine samples collected for peptidome analysis and association with GFR<60 mL/min 6 months post-LT	Very low (D)
	Results: Assessed thousands of peptides in the urinary peptidome, none associated with CKD at 6 months; Viral hepatitis sole independent predictor for CKD	
	Limitations: small sample; single center; single sample collection timepoint	

### Recommendation

Based on the very low quality of evidence available, the following recommendation was issued: We suggest that biomarker assays may be able to help predict chronic kidney disease after liver transplantation.

Quality of evidence: Very Low.

Strength of recommendation: weak for.

### Discussion and Next Steps

Given the high prevalence of CKD in post-LT patients, early identification of patients at risk for developing CKD is crucial for targeting interventions to prevent renal deterioration. Biomarkers such as uGAL, B2MG, CD40 antigen, and others may be helpful in the early identification of LT who are prone to developing CKD. However, the available data is insufficient for recommending a specific clinical protocol for using biomarkers to guide reno-protective interventions in post-LT patients. It is also unclear if collecting these biomarkers pre-transplant or post-transplant is more predictive of post-transplant CKD development. The limited number of studies assessing biomarkers for post-LT CKD mostly utilizes small single-center cohorts without independent validation cohorts, making their findings difficult to generalize to the broader LT population. Rather than utilizing single biomarker, it is possible that a combination of multiple biomarkers and clinical variables is the optimal strategy for predicting post-LT CKD. Further studies are needed to validate biomarkers for CKD prior to incorporating into post-LT clinical management including targeting patients for early CNIs reductions.

## Summary

LT is a complex medico-surgical process, the consequences of which are lifelong for recipients. While surgical and infectious complications commonly arise in the early post-LT period, the majority of more remote complications are related to disease recurrence and adverse effects of ongoing IS therapy (cancers, cardiovascular disease, and CKD, in particular). Traditionally, non-specific and oftentimes invasive monitoring has been needed to detect recurrent or *de novo* disease processes as well as to direct interventions, including the active reduction of IS therapy. In recent years, however, the focus of the transplant community at large has shifted to identifying more non-invasive biomarkers, in order to objectively measure and even predict the appearance of adverse events in transplant recipients.

Table 11 summarizes the specific research questions and recommendations formulated by this Working Group regarding the use of biomarkers in post-LT patient care. For studies evaluating use of biomarkers in predicting or detecting disease recurrence, including HCC, methodologies and findings are rather inconsistent, and evidence remains low. For these relatively rare post-LT events, future studies will require simultaneously recruiting patients at multiple centers and likely in different countries, in order to accrue a sufficient number to evaluate biomarker efficacy. For more routine post-LT care, use of biomarkers to tailor IS management appears helpful, but clear recommendations can still not be given regarding which specific marker or set of markers to use. In the future, larger studies including more diverse post-LT patient populations are needed to validate the utility of makers that have shown promise in preliminary clinical trials.

**TABLE 11:** Summary of research questions and recommendations.

Topic	Research question	Recommendation	Quality of evidence	Grade
Recurrent diseases	Can biomarkers be used to diagnose recurrent liver diseases after LT?	Additional studies are needed before a recommendation can be issued regarding the application of biomarkers to reliably predict and/or diagnosis disease recurrence after LT.	Very low	Strong for
Recurrent HCC	Can biomarkers be used to predict HCC recurrence?	While preliminary studies suggest a role for molecular biomarkers measured in liquid biopsy (circulating tumor cells, in particular), in prediction of HCC recurrence, additional studies are needed before any recommendation can be issued regarding their application in clinical practice, either as predictive factors to select patients for LT or to guide post-LT management	Low	Weak for
Immunosuppression weaning	Can biomarkers be used to safely wean immunosuppression?	Biomarker assays may be able to help guide immunosuppression weaning by monitoring liver injury. The use of longitudinal evaluations using non-invasive markers may lead to better stratification of patients for this purpose	Moderate	Weak for
Chronic kidney disease	Can biomarkers be used to predict chronic kidney disease in LT recipients?	Biomarker assays may be able to help predict chronic kidney disease after LT.	Very low	Weak for

## Figures and Tables

**Table:** 

AIH	Autoimmune Hepatitis
AFP	alpha-fetoprotein
AMA	anti-mitochondrial antibody
ANA	anti-nuclear antibodies
AR	acute rejection
ASMA	anti-smooth muscle antibodies
B2MG	beta-2 microglobulin
CET	Centre for Evidence in Transplantation
cfDNA	cell free DNA
CKD	chronic kidney disease
CNIs	calcineurin inhibitors
CRP	Serum C-reactive protein
CTCs	circulating tumor cells
DCP	des-gamma-carboxy prothrombin
ESOT	European Society of Organ Transplantation
HCC	hepatocellular carcinoma
HLA	human leukocyte antigen
IgG	immunoglobulin G
ILTS	International Liver Transplant Society
IS	immunosuppression
ISW	immunosuppression withdrawal
LDLT	live donor liver transplantation
LRT	locoregional therapy
LT	liver transplantation
MASH	metabolic dysfunction-associated steatohepatitis
MC	Milan criteria
PNPLA3	patatin-like phospholipase domain-containing protein 3
PICO	Population, Intervention, Comparator and Outcome
PBC	Primary biliary cholangitis
PSC	Primary sclerosing cholangitis
rAIH	recurrent AIH
rPBC	recurrent PBC
rPCS	recurrent PSC
US	United States
uNGAL	urinary neutrophil gelatinase-associated lipocalin.

## Data Availability

The original contributions presented in the study are included in the article/Supplementary Material, further inquiries can be directed to the corresponding author.
