# Supplementary material for: European Society for Organ Transplantation Consensus Statement on Biomarkers in Liver Transplantation
Source: Transpl Int. 2023 Aug 30;36:11358. doi: 10.3389/ti.2023.11358 (PMC10498996; doi:10.3389/ti.2023.11358)
Supplement: Supplementary file 1 [file DataSheet1.docx]

**Supplementary Table 1**. **Main characteristics of studies assessing biomarkers for recurrent diseases in liver transplantation.**

| **1^st^author/journal/date** | **Study type** | **No. of patients** | **Main outcomes assessed and results** |
| --- | --- | --- | --- |
| **AIH** | | | |
| Gonzalez-Koch, Liver Transplantation, 2001 | Observational non comparative retrospective | **41** | Frequency, risk factors, consequence of recurrence.  Recurrence in 7 (17%) patients. Recurrent autoimmune hepatitis more commonly had **HLA-DR3 or HLA-DR4** |
| Duclos-Vallée, Gut 2003 | Observational non comparative retrospective | **17** | Long-term outcome (10-year)  Recurrence in 7 (41%) patients.  **HLA DR3** |
| McCabe, Transpl Direct 2021 | Observational non comparative retrospective | **75** | Frequency, risk factors, consequence of recurrence.  Recurrence in 20 (27%) patients. High-level **HLA-DR mismatch** |
| **PBC** | | | |
| Sanchez, Transplantation, 2003 | Observational comparative retrospective | **156** | Recurrence  Recurrence 17 (10.9%). **Donor alleles A1, B57, B58, DR44, DR57, and DR58** were found at an increased frequency. The only **recipient** allele of significance was **B48**. Not any statistically significant difference in mis- matches. |
| Guy, Liver Transplantation, 2005 | Observation comparative retrospective | **48** | Risk factors for PBC recurrence.  Recurrence in 27 (56%) patients.  Increased mismatch of **donor DR3 and recipient DR4** in patients with recurrent PBC (p<0.055) |
| Morioka, Liver Transplantation, 2007 | Observation comparative retrospective | **50** | Patient survival and PBC recurrence in LDLT.  Recurrence 9. a **higher n**umber of **HLA-A, -B, and -DR mismatches** between donor and recipient  the presence of persistent ascites before LDLT, (donor age > 50 years) > survival. **Lower number of HLA mismatches** between donor and recipient, and a lower average trough level of tacrolimus within 1 year after LDLT > recurrence. |
| Carbone, AJT 2013 | Observation comparative retrospective | **248** | Risk loci for PBC in the native liver might influence the risk of PBC recurring.  Recurrence 105 (42.3%) **SNPs rs62270414** |
| **PSC** | | | |
| Alexander, Liver Transplantation 2008 | Observational comparative retrospective | **69** | Risk factors recurrence  Recurrence 7 (10%). Presence of **HLA-DRB1*08** **in recipient**  (+ Rejection episodes and steroid-resistant rejection) |
| Bajer, *World J Gastroenterol* 2018 | Observational non comparative retrospective | **47** | Risk factors recurrence  Recurrence 21.  **HLA-DRB1*07 in the donor** |
| **MASH** | | | |
| Finkenstedt, Clin Gastro and Hepatol 2013 | Observational non comparative retrospective | **237 recipients**  **95 with macrovesicular steatosis** | Association of donor and recipient risk alleles  Recipient who carried **rs738409-GG** in PNPLA3 had a 13.7-fold higher risk of graft steatosis |
| Mowry, Transpl Direct 2021 | Observational non comparative retrospective | **37** | MASLD vs MASH  Identification of 14 circulating metabolites characterizing MASLD (vs normal liver)  Identification of 16 circulating metabolites characterizing MASH (vs MASLD) |

*AIH=autoimmune hepatitis; LDLT= live donor liver transplantation; MASLD= metabolically associated steatotic liver disease; MASH= metabolically associated steatohepatitis; PBC= primary biliary cholangitis, PSC= primary sclerosing cholangitis*

**Supplementary Table 2. Characteristics of studies evaluating biomarkers for HCC recurrence.**

| **Reference** | **Design** | **N** | **Setting** | **Patient** | **Marker** | **Technique** | **D or V** | | **Primary outcome** | **Results** |
| --- | --- | --- | --- | --- | --- | --- | --- | --- | --- | --- |
| **Exosomal miRNA** | | | | | | | | | | |
| Nakano T. Am J Transpl (2019) 19:3250–62. doi: 10.1111/ajt.15490 | Retrospective | LT: 121  HCC 93  CLD 28 | Pre and post-LT  Extra-Milan  LDLT | Adults  All causes | Exosomal miRNA 92b | Microarray profiling  qRT-PCR | D | | Recurrence | - Post-LT, exosomal miR-92b level predicted early recurrence (AUC= 0.925, p < 0.001; sensitivity = 85.7%, specificity = 86.0%).  - Circulating exosomes impact on HCC development partly through suppression of CD69 on NK cells my hepatoma-derived exosomal miR-92b |
| Sugimachi K. Br J Cancer (2015) 112:532–8. doi: 10.1038/bjc.2014.621 | Case control | LT 65 | PreLT  Extra- Milan  LDLT | Adults  All causes | Exosomal miRNA  718, 1246 | Microarray profiling  qRT-PCR | V | | Recurrence  RFS | - Exosomal miR-718 and miR-1246, downregulated and upregulated, respectively, in recurrent vs non-recurrent patients.  - Low miR-718 expression associated with poorer histological differentiation (p = 0.026) and beyond Milan criteria status (p = 0.04). - Exosomal miR-718 expression not associated with RFS (p = 0.13). |
| **mRNA and MiRNA** | | | | | | | | | | |
| Cheung ST. Transplantation (2008) 85:81–7. doi: 10.1097/ 01. tp.0000298003.88530.11 | Retrospective | LT 82  HCC 72  CLD 10  HC 10 | PreLT  Milan up to year 2002  USCF  Year above 2002 | Adults  All causes | mRNA  Albumin | qRT-PCR | D | | Recurrence  RFS  OS | Pre-LT, high level of albumin mRNA (>14.6) was a prognostic factor of:  - Recurrence (HR, 5.9; 95% CI, 1.9–18.8; p = 0.002)  - OS (HR, 4.6; 95% CI, 1.6–13.8; p = 0.006)  - RFS (HR, 4.3; 95% CI, 1.6–11.8; p = 0.005). High plasma albumin mRNA level predicted 2-year HCC recurrence with sensitivity and specificity of 73% and 70%, respectively |
| Oya H. Transplant Proc (2006) 38:3636–9. doi: 10.1016/j.transproceed.2006.10.172 | Retrospective | LT 14 | Pre and post- LT  Milan & extra-Milan  LDLT | Adults  All causes | mRNA  h-TERT  AFP | RT-PCR | D | | RFS | Pre-LT h-TERT mRNA associated with RFS (p = 0.005) but not AFP mRNA (NS). RFS 🡪 No significant difference between those who met Milan and those who did not; and no difference among positive vs. negative AFP mRNA |
| Marubashi S. Transpl Int (2007) 20 :576– 82. doi: 10.1111/j.1432-2277.2007.00480.x | Prospective  Case control | LT 48 HCC 32  ESLD16  LDC 48 | Pre and post-LT  Milan and extra-Milan  LDLT | Adults  All causes | mRNA  AFP | qRT-PCR |  | | Recurrence | Pre-LT AFP mRNA: prognostic factor of recurrence (HR, 10.8; 95% CI, 1.53-76.9; p= 0.017) |
| Wang Y. Hepat Mon (2011) 11:195–9. | Retrospective  Case control | LT 49  HCC 29  CLD 20  HCC 20 | Pre and post-LT  Milan and extra-Milan | Adults  HBV and HCV | mRNA  AFP  GPC3 | RT-PCR | D | | Recurrence | - Pre-LT, AFP mRNA: prognostic factor of recurrence (RR, 2.91; 95% CI, 1.09– 7.76; p = 0.033). - Post-LT, AFP mRNA:not prognostic factor of recurrence (RR, 2.62; 95% CI, 0.93–7.41; p = 0.07).  - GPC3 mRNA level: not associated with recurrence. |
| Hwang HS. Gut Liver (2022) 16(3):443–455. doi: 10.5009/gnl210162 | Prospective  Case control | LT 25 | Pre and  Post-LT    Milan and downstage  LDLT | Adults  All causes | mRNA, K19  EpCAM  CD90  SNAIL  TWIST | qRT-PCR | D | | Recurrence  RFS | - EpCAM and CD90 mRNA levels correlated with the detection rate of EpCAM+ and CD90+ CTCs but no prognostic value.  EpCAM+/CD90+ CTC can be used preoperatively and 1 day after LDLT as markers iin LT selection and postLDLT management.  - mRNA levels of K19, SNAIL and TWISTnot associated with recurrence. |
| Huang A. J Cancer (2021) 12:7190–200. doi: 10.7150/ jca.59612 | Retrospective | LT 213  HCC  193  ESLD20 | Pre and post-LT  Milan & extra Milan | Adults  All causes | miRNA  miR 122, 192, 21, 223,26ª, 27ª, 801 | qRT-PCR | V | | Recurrence | - Positive miR panel status in the late phase (7–14 days): prognostic factor of recurrence (HR, 4.90; 95% CI, 2.20–10.95; p < 0.001). - mi-R panel: earlier predictor of recurrence than AFP and DCP. |
| Ng KT-P. Oncotarget (2016) 7:19824–39. doi: 10.18632/oncotarget.7627 | Retrospective | LT 62  HC 12 | Pre and post-LT  Milan and extra Milan | Adults  All causes | miRNA  miR 148ª, 1246, 1290, Let7c, 21,23b,27b,122,125b, 151-5p, 192,195,199a-3p, 215 |  | V | | Recurrence  RFS  OS | miR-148a (p=0.03) & miR (p=0.009): predictors of HCC recurrence. In the early phase (2-h after portal vein reperfusion), upregulation of miR-1246: prognostic predictor of both RFS (HR, 10.12; 95% CI, 1.45–70.47; p = 0.020) and OS (HR, 10.24; 95% CI, 1.39–75.67; p = 0.023). |
| **Circulating T cells** | | | | | | | | | | |
| Xue F. Oncol Lett (2018) 15:5481–8. doi: 10.3892/ ol.2018.8019 | Retrospective  Case control | LT 30  HCC 10 | Pre and post-LT  Milan & extra Milan | Adults  All causes | CTC  CEP8, CK+, DAPI+, CD45- | iFISH  CellSearch | V | | Recurrence  RFS | - Comparison of detection performance of iFISH® vs. CellSearch®: FISH® > CellSearch® (sensitivity 70% vs. 26.7%; p < 0.01). Threshold >5/7.5ml - Pre-LT iFISH® CTC count predicted recurrence on univariable analysis (HR, 5.14; 95% CI, 1.53-17.31; p = 0.008). |
| Wang P-X. Liver Int (2021) 41:562–73. doi: 10.1111/liv.14734 | Retrospective | LT 193 | Pre and post-LT | Adults | CTC  EpCAM+, Pan-CK+, CK19+, DAPI+, CD45- | ChimeraX il20  Single-cell WGS |  | | Recurrence | - Pre-LT CTC count: low predictive value for recurrence - post-LT CTC count: prognostic factor for recurrence (HR, 2.67; 95% CI, 1.51–4.74; p = 0.001). |
| Chen Z. Ann Transl Med (2020) 8:1067. doi: 10.21037/atm-20-2751 | Retrospective | LT 50 | PreLT  Milan & extra Milan | Adults  All causes | CTC  CEP8  DAPI+  CD45- | Negative enrichment and imFISH | D | | Recurrence  RFS  OS | Pre-LT CTC count: prognostic factor for recurrence (RR, 5.41; 95% CI, 1.13–25.87; p = 0.034). Threshold >1/3.2ml 1-year RFS rate of CTC-neg and CTC -positive patients: 91.7 and 61.5% respectively (p.=0.02). 1-year OS of CTC-positive and CTC-neg: 88.5% and 91.7%, respectively (p=0..751) |
| Wang S. J Investig Med (2018) 66:1–6. doi: 10.1136/jim-2017-000655 | Prospective | LT 47 | Pre and post-LT  Milan & Extra Milan | Adults  Only HBV | CTC,  EpCAM+, CK8+, CK18+, CK-19+, DAPI+, CD45-, Vimentin+, Twist+ | CanPatrol  RNA-ISH | D | | Recurrence | - Three different subtypes of CTCs identified: epithelial, interstitial &mixed. - post-LT, changes in the proportion of CTCs subtypes (increased epithelial and interstitial CTC levels). - CTC count and subtypes not predictive of recurrence (p > 0.05). |
| Xie Y-L. Asian J Surg (2022) 45:435–40. doi: 10.1016/j.asjsur.2021.07.058 | Retrospective | LT 56 | Pre and post-LT  Milan & Extra Milan | Adults  All causes | CTC  EpCAM+, CK8+, CK18+, CK19+, Vimentin+, twist+ | CanPatrol  RNA-ISH | | D | Recurrence | - Three different subtypes of CTCs identified: epithelial, interstitial, and mixed. Interstitial CTCs showed particular interest. -Perioperative increasing proportion of interstitial CTC: prognostic factor of recurrence (HR, 6.17; 95% CI, 1.89–20.18; p = 0.003). |
| Hwang HS. Gut Liver (2022) 16(3):443–455. doi: 10.5009/gnl210162 | Prospective | LT 25 | Pre and post-LT  Milan & Extra-Milan  LDLT | Adults  All causes | CTC  EpCAM+  CD90+  CD45- | Fluorescenc  Activated cell sorting | | D | Recurrence  RFS | - Three different subtypes of CTCs identified: EpCAM+ (epithelial), CD90+ (mesenchymal) and EpCAM+/CD90+ (mixed). - Pre-LT, EpCAM+ CTC count associated with lower RFS (p = 0.025). - Detection of EpCAM+/CD90+ CTCs on POD 1: prognostic factor of recurrence (HR, 26.88; 95% CI, 1.86–387.51; p = 0.016). |

*CC=case control; CLD=chronic liver disease; CTC=circulating T cells; D= discovery; LDLT=live donor liver transplantation; LT=liver transplantation; D=discovery; P=prospective, POD= postoperative day; R=Retrospective; RFS= recurrence free survival; V=validation; WGS=whole genome sequencing*

**Supplementary Table 3. Characteristics of the studies assessing the role of biomarkers for IS minimization and/or withdrawal.**

| **Author, Date** | **Journal** | **Study design** | **Cohort (Clinical Trial)** | **Setting** | **Clinical/**  **Biological Marker** | **Invasive or Noninvasive** | **No. of patients in ITT** | **Discovery/**  **Validation** | **Main outcome assessed** | **Additional Notes** |
| --- | --- | --- | --- | --- | --- | --- | --- | --- | --- | --- |
| **Weaning IS without rejection, time to minimal/no immunosuppression** | | | | | | | | | | |
| Appenzeller-Herzog, 2021 | Am J Trans | Systemic review (adult LT) | Barcelona/Rome/  Leuven  Pamplona  Murcia 1  Murcia 3  Chicago | Non-autoimmune/ non-replicative viral (NINV) Adult LT recipients | (**Non-invasive**)  Time between LT and ISW; Lower lymphocyte proliferation; PBMC cell and gene expression; Serum ferritin. (**Invasive**) Gene expression in allograft (*CDHR2, MIF, PEBP1, SOCS1,* *TFRC*); Intrahepatic CD4+ Cells; Hepatocytic iron | Both | Varies based on cohort and study | Varies | Predisposition to ISW |  |
| Chruscinski A, 2022 | Clin Exp Immunol | Prospective  observational single-center, case-control | LITMUS Phase 2a  Single center  (NCT02541916) | Adult LT recipients with normal liver function 3 months post-transplant | (**Non-invasive**)  PBMC GEX: FGL2/IFNG ratio  (**Invasive**) Intrahepatic GEX: FOXP3/IFNG ratio | Both | Phase I: Enrollment, n = 69  Biomarker positive, n = 28 Phase II, n = 14  Tol, n = 8 non-Tol, n = 6 | No comparison | Predisposition to ISW | IS withdrawal done over 3-4 months |
| Vionnet J, 2021 | J Hepatol | Retrospective cross-sectional study | LIFT  (NCT024989977*) | 3 years post-transplant and had normal liver function tests at the screening visit | **Intrahepatic GEX:**  Probability of TCMR-for subclinical graft injury  **Non-Invasive:**  ALT/LSM ratio  ALT/class II DSA cMFI ratio | Both | LIFT  Total enrolment: n=190  IS minimization: n=122  +  iWITH, n=157 | Yes  Discovery = LIFT  Validation = iWITH | Subclinical graft injury |  |
| Jucaud V, 2019 | Hepatology | Prospective cohort | ITN030ST trial | Patients were randomized to stable IS monotherapy following which they were weaned off IS | dnDSA | Non-invasive | Cohort, n=69  [disqualified, n=12; Excluded, n=17]  Completed study, n=40  [IS maintenance (control), n=9;  Tol, n=9; non-Tol, n=22] | No | Acute rejection during ISW | IS withdrawal was done over a 6–9-month period |
| Shaked A, 2017 | Hepatology | Retrospective study | ITN030ST | Recipients in both studies were followed from transplantation for up to 48 months after surgery. | serum miRNA  (hsa-miR-483-3p and hsa-miR-885-5p) | Non-invasive | Discovery set (ACR, n = 18; non-ACR, n = 45) and validation set (ACR, n = 19; non-ACR, n = 48) | differentiated ACR from non-ACR training set with an AUC of 90% (95% CI = 81%-98%), with 88.9% sensitivity and 83.3% specificity in the (P = 0.0001) test set with an AUC of 89% (95% CI = 79%-98%), with 84.2% sensitivity and 85.4% specificity | Subclinical graft injury prediction | This was for ACR identification |
| Shaked A, 2017 | Hepatology | Retrospective study | ITN030ST | Recipients in both studies were followed from transplantation for up to 48 months after surgery. | serum miRNA | Non-invasive | Patients with ISW, n=27;  (Longitudinal assay;  Samples tested: ACR, n = 64; pre-ACR, n=45 post-ACR, n=19.  Tol, n = 55: pre-biopsy, n=38; post-biopsy, n=17 | miRNA ACR diagnostic test separated ACR events from non-ACR events, with 95% CIs of the smoothed means for these two groups separating at approximately 40 days prior to the biopsy-proven rejection | Subclinical graft injury prediction |  |
| Taubert R, 2016 | Am J Trans | Retrospective longitudinal study | NCT00647283 | multicenter trial of IS withdrawal. | Association between portal vein infiltrates and elapsed time post-ISW  Treg (CD4+FOXP3+) portal infiltration at 1-yr post ISW.  **Intrahepatic GEX:**  *FOXP3,* *CXCL10*, *CXCL9*, *UBD*, *IRF1*, *STAT1*  *IL32*, *CD52*, *CD68*, *STAT1*, *GPNMB, S1PR1, RGS5*, *ENPP2*, *MSL3*, *OPN3*, *PAK2*, *CDH5*, *SELP* | Invasive | Spontaneous Operational Tolerance patients, n = 24  (n = 18 for immunohistology)  (n = 12 of 18 for flow cytometry)  (n = 17 for transcriptional analyses)  Additional, n=14, non-Tol recipients before IS withdrawal and while undergoing rejection | No | IS withdrawal with no rejection | IS doses were gradually decreased until complete discontinuation over 6–9 months |
| de la Garza, 2015 | Transplant Immunol | prospective study | Navarra | Adult LT recipients | Secretion of cytokines by PBMCs in culture | Non-invasive | Enrolled in trial, n=31  Enrolled in study, n=24  Tol, n=15  Non-Tol, n=9 | Not done | Response to ISW  Graft Injury during ISW | IS therapy was divided into 6 to 10 fractions, and gradually reduced every month until complete withdrawal or rejection |
| Garcia MJ, 2015 | Trans Proceedings | Cross-sectional | Santander | Adult LTR with stable function for 8 years | Galectin-1 | Non-invasive | Enrolled in study, n=80  LTR with IS and stable function, n=30  LTR with ARE, n=15  LT without ARE, n=35 | Not done | Acute graft injury | The LTR has a higher number of patients with cardiac complication, a direct relation between cardiomyopathy has galectin-1 has been demonstrated. Could this be influencing the outcome? |
| Bohne F, 2014 | Sci Translational Med | Prospective study | Barcelona/Valencia  (NCT00668369) | Adult LT recipients with detected HCV RNA | Blood Vδ1/Vδ2 T cell ratio | Non-invasive | Total cohort, n=32  Tol, n=17  Non-Tol, n=15 | Yes,  n=67 | ISW and graft acceptance in HCV+ positive patients |  |
| Benitez C, 2013 | Hepatology | prospective cohort | Barcelona/Rome/Leuven | adult stable liver transplant recipients | Time (mo) from transplant  gender  age at transplantation  calcineurin inhibitors in the IS drug regimen. | Clinical | Enrolled, n = 102  Tol, n = 41  Non-Tol, n = 57  [withdrawn from the study during dose minimization, n = 4] | No | Acute rejection during IS withdrawal.  Safety of ISW | Drugs were gradually discontinued over a 6 to 9-month period.  Tolerance was independently associated with time since transplantation (odds ratio [OR] 1.353; P = 0.0001), recipient age (OR 1.073; P = 0.009), and male gender (OR 4.657; P = 0.016) |
| de la Garza, 2013 | Liver Trans | prospective study | Navarra | Adult LT recipients | Time from transplantation  Stimulation lymphocyte reactivity index | Non-Invasive | Enrolled in trial, n=31  Enrolled in study, n=24  Tol, n=15  Non-Tol, n=9 | No | Response to ISW  Graft Injury during ISW | Same population as de la Garza, 2015 |
| Bohne F, 2011 | JCI | Prospective multicenter | Barcelona/Rome/Leuven | Adult stable LT recipients | **Intrahepatic GEX**  5-gene (*CDHR2*, *MIF*, *PEBP1*, *SOCS1*, *TFRC*) signature  Iron metabolism genes, *HAMP* and *TFRC* (FDR = 0, FC > \|2\|), and FTHL12 and FTHL8 | Invasive | Enrolled in trial, n = 102  Enrolled in study, n = 75  Discovery cohort, (Barcelona)  Tol, n = 20  Non-Tol, n = 28  Validation set.  (Rome and Leuven)  Tol, n = 10  Non-Tol, n = 11 | No | Predisposition to ISW | Immunosuppressive drug doses were gradually decreased until complete discontinuation over 6–9 months.  (Same patient population as Benitez C, 2013)  Additional iron homeostasis-associated genes were differentially expressed at FDR=25. |
| Girinita A, 2010 | Human Immunol | Retrospective study | Pittsburg | Pediatric and adult LTRs. | Anti-HLA Abs | Non-Invasive | 73 clinically stable liver transplant recipients divided into group A (n = 19; clinically tolerant), group B (n = 34; undergoing weaning, on minimal immunosuppression), and group C (n = 20; had failed drug withdrawal or weaning never attempted). | No | Predisposition to ISW |  |
| Millán O, 2010 | Clinical Immunol | Prospective case-control study | Barcelona-2 | Adult Cadaveric donor LTR | IFN-γ  PBMC Profile  %CD8+IL-2  %CD8+IFN  %CD4+IFN | Non-invasive | Enrolled, n=24  Tol, n=13  Non-Tol, n=11  [rejection, during ISW, n=7;  After ISW complete, n=4] | No | Indicator of acute rejection with ISW | IS doses were gradually reduced over 6–9 months |
| Martinez-Llordella M, 2008 |  | Prospective study |  | Adult | PBMCs GEX | Non-invasive | Tol, n = 28  non-Tol, n = 33 |  | ISW |  |
| Tisone G, 2006 |  | Prospective study |  | HCV–RNA positive LT patients under cyclosporine A monotherapy | Time after transplant,  Patients being treated with ribavirin.  cyclosporine A trough levels  Initial steroid-free immunosuppression | Both | 34 patients  Tol, n = 8  non-Tol, n = 26  [Rejection, n = 12.  Rejection after complete withdrawal, n = 14] |  | Predisposition to ISW  Response to ISW  HCV positive LTRs |  |
| **Genomics and molecular-based markers for acute graft injury following liver transplantation** | | | | | | | | | | |
| Cox DRA, 2022 | Liver Trans | Prospective Study | Austin Health -2 | Adult Tx recipients | Hepatocyte-Specific methylated cell-free DNA | Non-invasive | Enrolled, n = 51  [no rejection, n = 14; rejection, n = 37 (severe, n = 4; moderate, n = 12; mild, n = 12; intermediate, n = 9)] | No |  |  |
| Levitsky J, 2021 | Am J Transplant | Prospective Study | NU and CTOT-14 | Adult first LTx for acute rejection | Donor-derived cell-free DNA | Non-invasive | Enrolled, n = 219  (enrolled subjects were split, Discovery:Validation, 70:30)  [AR, n = 57 (NU, n = 43; CTOT-14, n = 14), ADNR, n = 68 (NU, n = 41; CTOT-14, n = 27), and TX, n = 94 (NU, n = 47, CTOT-14, n = 47)] | Yes |  | The study also funded in part by Eurofins-Viracor |
| Goh SK, 2019 | Trans Direct | Prospective Study | Austin Health | Adult brain-dead LTx recipients | Donor-derived deletion/insertion polymorphisms in cell-free DNA | Non-invasive | Longitudinal cohort, n = 20  (Uneventful, n = 14; tBPAR, n = 3; cholestasis, n = 3)  Cross-sectional cohort, n = 20  (No tBAR, n = 10; tBAR, n = 10) | Yes | Differentiating between AR and normal LTx phenotypes | tBAR, treated Biopsy-Proven Acute Rejection  Requires prior knowledge of deletion/insertion polymorphism (DIP) from the tested 9 sites. ddPCR used.  They do not talk about running controls and assessing the differences that the assay can detect |
| Millán O, 2019 | Front Immunol | Prospective Study | Barcelona-3 | Adult Tx recipients | Plasma signature of miR-181a-5p | Non-invasive | Enrolled, n = 145  (Non-rejectors, n = 120; rejectors, n = 17; subclinical rejectors, n = 8) | No | Identification of TCMR risk |  |
| Schütz E, 2017 | PLOS Med | Prospective Study | Germany-multicenter | Adult LTx recipients | Donor-derived cell-free DNA | Non-invasive | Enrolled, Patients (N) = 107; Samples collected (n) =  (Stable, N = 88, n = 393; ) | No | Differentiating between AR and normal LTx | Requires prior knowledge of SNPs present from the tested 40 SNPs. ddPCR used. They do not talk about running controls and assessing the differences that the assay can detect |

*ACR= acute cellular rejection; AR= acute rejection; IS= immunosuppression; ISW=immunosuppression withdrawal; ITT= intention to treat; LTx=liver transplantation; LTR=liver transplant recipients; PBMC= peripheral blood mononuclear cells; tBAR, treated Biopsy-Proven Acute Rejection; Tol=tolerant; TCMR= T-cell mediated rejection; Tx=transplantation*

**Supplementary Table 4. Characteristics of studies assessing the role of biomarkers in predicting posttransplant CKD.**

| **Paper** | **Study Design** | **Number of centers** | **N** | **Setting** | **Biomarkers assessed** | **Discovery/Validation** | **Outcomes Assessed** |
| --- | --- | --- | --- | --- | --- | --- | --- |
| Levitsky 2020 | Prospective discovery and validation cohort | Multicenter (8 centers) | n=110 (multicenter discovery cohort n=60; single center validation cohort n=50) | Samples collected 3 months post-LT in patients with preserved GFR | Beta-2 microglobulin (B2MG), CD40 antigen | Discovery and Validation cohorts | Glomerular filtration rate (GFR) deterioration at year 1 and year 5 in liver transplant (LT) recipients |
| Cullaro 2018 | Retrospective assessment of prior prospective cohort study | Single center | n=92 | Adults with LT from 2008-2010 | urinary neutrophil gelatinase-associated lipocalin (uNGAL) | Discovery | post-LT CKD (GFR<60 mL/min for 3 months); median follow-up 4.6-5.1 yrs; |
| Levitsky 2011 | Retrospective identification with prospective proteomic assays | Single center | n=342 | baseline GFR>60 mL/min at time of transplant and now >3 years post-LT | Cyc, alpha-1-microglobulin, beta-2-microglobulin, TFF3, FABP, factor VII, apolipoprotein H, apolipoprotein CIII, chromogranin A, CD40 | Discovery | degree of post-LT CKD (GFR>60, 60-90, and <60 mL/min) |
| Milongo 2015 | Prospective cohort | Single center | n=80 | pre-LT patients with GFR>60 ml/min; followed for 6 months post-transplant | thousands of peptides in the urinary peptidome | Discovery | Reduction in GFR to <60 mL/min at 6 months post-LT |

*CKD= chronic kidney disease; GFR=Glomerular filtration rate; LT=liver transplantation; Yrs= years*
